# Mechanisms of lymphoid depletion in bowel obstruction

**DOI:** 10.3389/fphys.2022.1005088

**Published:** 2022-09-21

**Authors:** You-Min Lin, Shrilakshmi Hegde, Yingzi Cong, Xuan-Zheng Shi

**Affiliations:** ^1^ Department of Internal Medicine, University of Texas Medical Branch, Galveston, TX, United States; ^2^ Department of Pathology, University of Texas Medical Branch, Galveston, TX, United States; ^3^ Department of Microbiology and Immunology, University of Texas Medical Branch, Galveston, TX, United States

**Keywords:** intestinal obstruction, mechanical stress, osteopontin, corticosterone, immune dysfunction, gut microbiome, lymphoid depletion

## Abstract

**Background and aims:** Bowel obstruction (BO) causes not only gastrointestinal dysfunctions but also systemic responses such as sepsis, infections, and immune impairments. The mechanisms involved are not well understood. In this study, we tested the hypothesis that BO leads to lymphoid depletion in primary and peripheral lymphoid organs, which may contribute to systemic responses. We also sought to uncover mechanisms of lymphoid depletion in BO.

**Methods:** Partial colon obstruction was induced with a band in the distal colon of Sprague-Dawley rats, and wild-type and osteopontin knockout (OPN^−/−^) mice. Obstruction was maintained for 7 days in rats and 4 days in mice. Thymus, bone marrow, spleen, and mesenteric lymph node (MLN) were taken for flow cytometry analysis.

**Results:** The weight of thymus, spleen, and MLN was significantly decreased in BO rats, compared to sham. B and T lymphopoiesis in the bone marrow and thymus was suppressed, and numbers of lymphocytes, CD4^+^, and CD8^+^ T cells in the spleen and MLN were all decreased in BO. Depletion of gut microbiota blocked BO-associated lymphopenia in the MLN. Corticosterone antagonism partially attenuated BO-associated reduction of lymphocytes in the thymus and bone marrow. Plasma OPN levels and OPN expression in the distended colon were increased in BO. Deletion of the OPN gene did not affect splenic lymphopenia, but attenuated suppression of lymphopoiesis in the bone marrow and thymus in BO.

**Conclusions:** BO suppresses lymphocyte generation and maintenance in lymphoid organs. Mechanical distention-induced OPN, corticosterone, and gut microbiota are involved in the immune phenotype in BO.

## Introduction

A homeostatic immune system requires normal lymphopoiesis in the primary lymphoid organs and maintenance of the lymphocyte populations in the peripheral lymphoid tissues. T and B lineage lymphopoieses occur mainly in the thymus and bone marrow, respectively in adults. Disruption of lymphopoiesis leads to acute thymus atrophy or deficiency of B cell lymphopoiesis, which decreases the body’s ability to maintain the peripheral lymphocyte repertoire and attenuates immunity to antigens (Hardy et al., 2000; Gruver and Sempowski, 2008). The maintenance of lymphocyte repertoire in the periphery is controlled by the interactions of the lymphocytes with the lymphoid tissues and many other neural and humoral factors and antigens (Goldrath and Bevan, 1999; Thome and Farber, 2015). When such control mechanisms are affected, lymphoid depletion may occur in the peripheral lymphoid tissues. Disruption of lymphocyte generation and maintenance causes immune impairments and severe health problems.

Obstructive bowel disorders (OBD) are characterized by lumen distention due to mechanical or functional bowel obstruction (BO) in the gut and present significant health challenges in adults and children (Welch and Welch, 1990; Shi and Terry, 2022). Current investigations focus mainly on the local dysfunctions in the gut, i.e., dysmotility, abdominal pain, and altered secretion and absorption functions (Roeland and von Gunten, 2009; Shi et al., 2011; Lin et al., 2017; Shi et al., 2018). However, bowel obstruction may lead to severe systemic responses, such as sepsis, septic shock, immune dysfunction, recurrent inflammation, and infections (Welch and Welch, 1990; Catena et al., 2019; Shi and Terry, 2022). In fact, most of the BO-related deaths (236,000 annually in the world) are due to systemic responses (Cappell and Batke, 2008; Catena et al., 2019). What accounts for the systemic responses in OBD is not well understood. Nevertheless, an impaired immune function has been reported in chronic OBD (Forchielli et al., 1997; Gosain et al., 2015). According to Forchielli et al. (1997), hypogammaglobulinemia is present in 78% of patients with chronic intestinal pseudo-obstruction. Immune abnormalities were also observed in patients with intestinal atresia (Notarangelo, 2014) and animal models of Hirschsprung’s disease (Frykman et al., 2015; Gosain et al., 2015). In these obstructive disorders, the primary pathologies are developmental anomalies either in the bowel wall (i.e., intestinal atresia) or enteric nervous system (i.e., Hirschsprung’s disease). However, these developmental anomalies lead to obstruction of the affected intestinal or colonic segments and consequently distention of the bowel segments proximal to the obstruction. Although immune impairments have been reported in these rare hereditary conditions or idiopathic OBD, the mechanisms and significance of immune impairments in these disorders are unknown. It was suggested that immune abnormality might be a cause of the disorders or a part of the disease process (Forchielli et al., 1997; Gosain et al., 2015). In this study, we wanted to test the hypothesis that persistent lumen distention in chronic bowel obstruction represents a unique pathological condition that may lead to immune abnormality by disrupting lymphopoiesis in the primary lymphoid organs and/or maintenance of lymphocyte populations in peripheral lymphoid tissues. If so, these changes may account for infections and other systemic responses encountered in OBD.

Here, we have used a well-established model of mechanical colon obstruction (Shi et al., 2011; Lin et al., 2017) to determine if BO-associated lumen distention may lead to immune organ atrophy and lymphoid depletion. It is found that partial colon obstruction in rats leads to marked thymus atrophy. Moreover, the numbers of B cells in the bone marrow, T cells in the thymus, and total, CD4^+^, and CD8^+^ T cells in the peripheral lymphoid organs including the spleen and mesenteric lymph node (MLN) are all dramatically decreased in BO. Our findings suggest that it is the mechanical distention in the gut that leads to immune impairments. Thus, BO may represent a model of lymphoid depletion. We have also sought to investigate mechanisms of BO-associated lymphoid depletion, particularly the role of microbiota, stress hormones, and mechano-sensitive gene expression of pro-inflammatory mediators in the immune phenotype.

## Materials and methods

### Animal models of partial colon obstruction, and pharmacological and antibiotics treatments

To induce partial colon obstruction, Sprague-Dawley rats, male and female of 8–10 weeks old, weighing 200–275 g (Harlan Sprague Dawley, Indianapolis, IN), were used in the study. The rats were housed in a controlled environment (22°C, 12-h light-dark cycle) and allowed food and water ad lib. The Institutional Animal Care and Use Committee at the University of Texas Medical Branch approved all procedures performed on the animals.

The rat model of partial colon obstruction was prepared as previously described (Shi et al., 2011; Lin et al., 2017; Hegde et al., 2018). Rats were anesthetized with 2% isofluorane inhalation by an E-Z Anesthesia vaporizer (Palmer, PA). After midline laparotomy, a distal colon segment 4 cm proximal to the end of the colon was carefully exposed. A small mesenteric window (3 × 3 mm^2^) was made next to the exposed colon segment. Partial colon obstruction was induced by placing a 3-mm wide medical grade silicone band around the colon wall through the small mesenteric window. The size of the silicon ring (21 mm in length) was 1–2 mm greater than the outer circumference of the colon when the colon segment was filled with fecal pellets, allowing a partial obstruction. The procedure to implement the silicon ring was completed within 2 min. The sham control rats underwent the same surgical procedure except that the ring was removed immediately after the 2-min procedure. Sham-operated and obstructed rats were euthanized 7 days after the operation.

In some experiments, sham and BO rats were administered with corticosteroid receptor antagonist RU486 (10 mg/kg, s.c, daily) (Choudhury et al., 2009; Zheng et al., 2013) starting on the day laparotomy was operated.

To eradicate gut microbiota, some sham and BO rats were treated with daily oral gavage of 1 ml of antibiotic cocktail solution as previously described (Hegde et al., 2018), starting 1 day before the laparotomy. The antibiotic cocktail consisted of metronidazole, ampicillin, and kanamycin each at 100 mg kg^−1^ body weight, and vancomycin at 50 mg kg^−1^ as described (Hegde et al., 2018).

Wild-type (WT) and OPN^−/−^ mice, male and female, 8-10 weeks old [Jackson Lab, 004936—B6.129S6(Cg)-Spp1tm1Blh/J], were also used to test the effect of BO on lymphoid organs. Partial colon obstruction in mice was surgically induced as previously described (Shi et al., 2011; Wu et al., 2013). The obstruction band used for mice was 2 mm wide and 10 mm long. The medical-grade silicone band was implanted around the distal colon, approximately 2 cm from the anus. Sham control mice were operated on, but the silicon band was removed immediately after being implanted. The mice were euthanized 4 days after the operation.

### Lymphoid tissue preparation and cell counts

Tissue samples of the thymus, spleen, and mesenteric lymph node (MLN) at the ileo-colon junction and the right thigh bone were taken from animals. The thymus, spleen, and MLN tissues were immediately placed in 2% FBS DMEM medium (Gibco, Grand Island, NY), and dissected free of fat. The solid tissues were weighed. Single-cell suspensions were prepared from the tissues by mechanical disruption as previously described (Cheng et al., 2011; Feng et al., 2011). In brief, tissue samples from the entire thymus, spleen, and MLN were minced and ground with syringe tips (23G) and filtered through a sterile 40 µm nylon cell strainer (Fisher Scientific, Hampton, NH). The bone marrow was processed into a single cell suspension by repeatedly flushing. Red blood cells (RBC) were removed with ACK buffer (NH_4_Cl 8.024 g/L, KHCO_3_ 1001 g/L, EDTANa_2_ ·2H_2_O 2.722 g/L). Cells were washed and re-suspended in flow cytometry staining buffer (PBS/2 mM EDTA/2% FBS), counted using a hemocytometer (Countess II, Life Technologies), and adjusted to 1 × 10^6^ cells/100 µl for flow cytometry.

### Surface marker analysis of lymphocytes by flow cytometry

Flow cytometry studies of the single cell suspensions were carried out with a BD LSRII-Fortessa™ system as previously described (Feng et al., 2011). The cells (100 µl of cell suspension including 10^6^ cells) were blocked for 5 min at room temperature (with mouse serum for rat cells, or rat-anti-mouse CD16/32 for mouse cells). Cells were then incubated on ice for 30 min with an antibody panel. For rat cells, the following antibody panels were used: CD3 (clone 1F4 BV421-labeled, 1/500, for total T cells), CD4 (clone OX-38, FITC-labeled, 1/500, for CD4 T cells), CD8a (clone OX-8; PE-labeled, 1/500, for CD8 T cells), and CD45RA (clone OX-33; BV786-labeled, 1/500, for B cells) (all from BD Pharmingen, San Diego, CA). Ghost Dye™ Red 780 was used to detect the live and dead cells (Tonbon Bioscience, San Diego, CA). Cells were then washed once with fresh buffer (PBS/2 mM EDTA/2% FBS) and ran flow cytometry freshly. For mouse samples, anti-CD3-APC (for total T cells), anti-CD4-FITC (for CD4^+^ T cells), anti-CD8a-PE (for CD8^+^ T cells), anti-CD45RA-BV421 (for B cells) (all from BioLegend) were used. Flow cytometry data were analyzed with FlowJo software (Ashland, OR). Cell numbers were calculated from the frequency of each positive staining against the total cell number in the sample.

### Colon tissue collection

Rat colon segments (each 1–2 cm in length) 2 cm proximal and 1 cm distal to the site of obstruction band, respectively, were collected in fresh carbogenated Krebs buffer (in mmol/l: 118 NaCl, 4.7 KCl, 2.5 CaCl2, 1 NaH2PO4, 1.2 Mgcl2, 11 days-glucose, and 25 NaHCO3). The segments were cleansed, opened along the mesenteric border, and pinned flat in a Petri dish with Sylgard base. The mucosa/submucosa (M/S) and muscularis externa (ME) layers were separated by microdissection as described previously (Shi and Sarna, 2008; Shi et al., 2011). The tissue samples were stored at −80°C for RNA or protein extraction and molecular studies.

### RNA preparation and real-time PCR

Total RNA was extracted from the ME and M/S tissues using the Qiagen RNeasy kit (Qiagen, Valencia, CA). One microgram of total RNA was reverse transcribed by using the SuperScript III First-Strand Synthesis System (Invitrogen) for real-time quantitative PCR (Lin et al., 2012; Lin et al., 2014). Real-time PCR was performed by use of the Applied Biosystems 7,000 real-time PCR system (Foster City, CA). The TaqMan primers and probes for detection of rat OPN mRNAs were purchased from Invitrogen. For relative quantitation of gene transcription, real-time PCR was performed with 40 ng cDNA for the target genes and the endogenous control (18 S rRNA). The cycling parameters for real-time PCR were as follows: uracil N-glycosylase activation at 50°C for 2 min, AmpliTaq activation at 95°C for 10 min, denaturation at 95°C for 15 s, and annealing/extension at 60°C for 1 min (repeat 40 times) on ABI7000. Duplicate cycle threshold (CT) values were analyzed in Microsoft Excel by the comparative Ct (ΔΔCT) method, as described by the manufacturer. The amount of target was obtained by normalization to endogenous reference 18 S rRNA.

### Enzyme immunoassay of plasma samples

Blood samples were collected from the left ventricle of animals immediately after euthanizing the animals. Plasma was collected and stored at -80°C. The levels of corticosterone and osteopontin in plasma samples were determined in enzyme immunoassay (EIA) as described (Shi and Sarna, 2008; Lin et al., 2012) with corticosterone and norepinephrine EIA kits (Cayman Chemical, Ann Arbor, MI) and osteopontin EIA kit (Enzo, Farmingdale, NY), respectively according to the manufacturers’ instruction.

### Statistical analysis

Data were expressed as mean ± SEM. Statistical analysis was performed by analysis of variance with nonrepeated measures by Student–Newman–Keuls test for comparisons of multiple groups, or by Kruskal–Wallis test followed by Dunn’s for nonparametric multiple comparisons, if data in a group does not follow a normal distribution. The Student’s t-test was used for comparisons of two groups. A *p* value of ≤0.05 was considered statistically significant.

## Results

### Colon obstruction led to atrophy of the thymus and peripheral lymphoid organs

**FIGURE 1:**
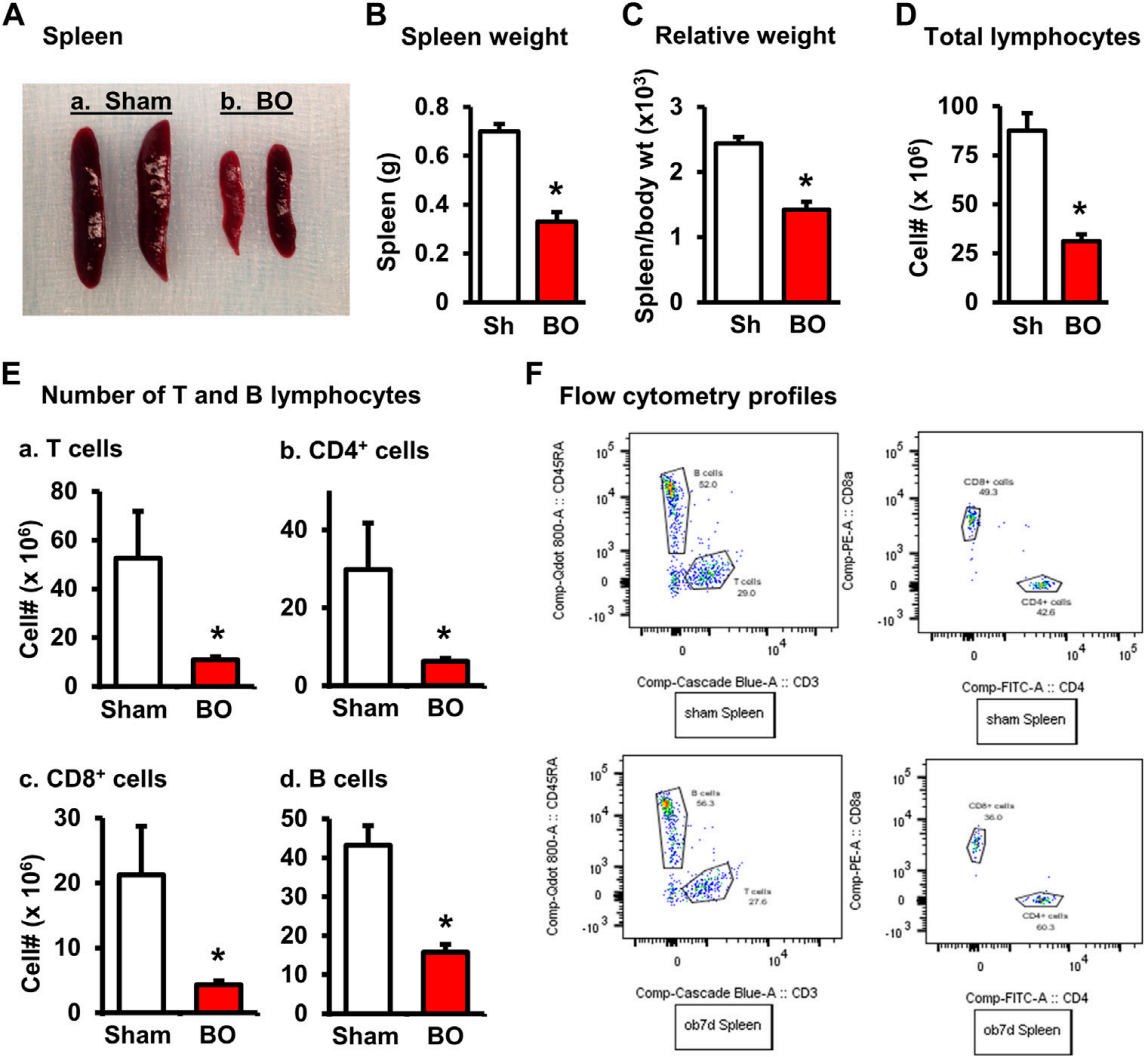
Effect of bowel obstruction on the spleen. **(A)** Isolated spleens from 2 sham rats (Sh) and 2 rats with bowel obstruction (BO) for 7 days. **(B)** Weight of spleens in Sh and BO rats. **(C)** Relative weight of the spleen [ratio of spleen weight/body weight (x 10^3^)]. **(D)** Total live cells. **(E)** Numbers of T and B lymphocytes in the spleen. **(A)**. Total T cells (CD3^+^); **(B)**. CD4^+^ T cells (CD3^+^/CD4^+)^; **(C)**. CD8^+^ T cells (CD3^+^/CD4^+^); **(D)**. B lymphocytes (CD45RA^+^). **(F)** Representative flow cytometry profiles for B and T lymphocytes in sham (upper left) and BO rats (down left), and CD4^+^ and CD8^+^ cells in sham (upper right) and BO rats (down right). N = 9 per group for B and **(C)**. N = 5 or 6 per group in D and **(E)**. *p < 0.05 vs. sham. Sh, sham. BO, bowel obstruction.

**FIGURE 2:**
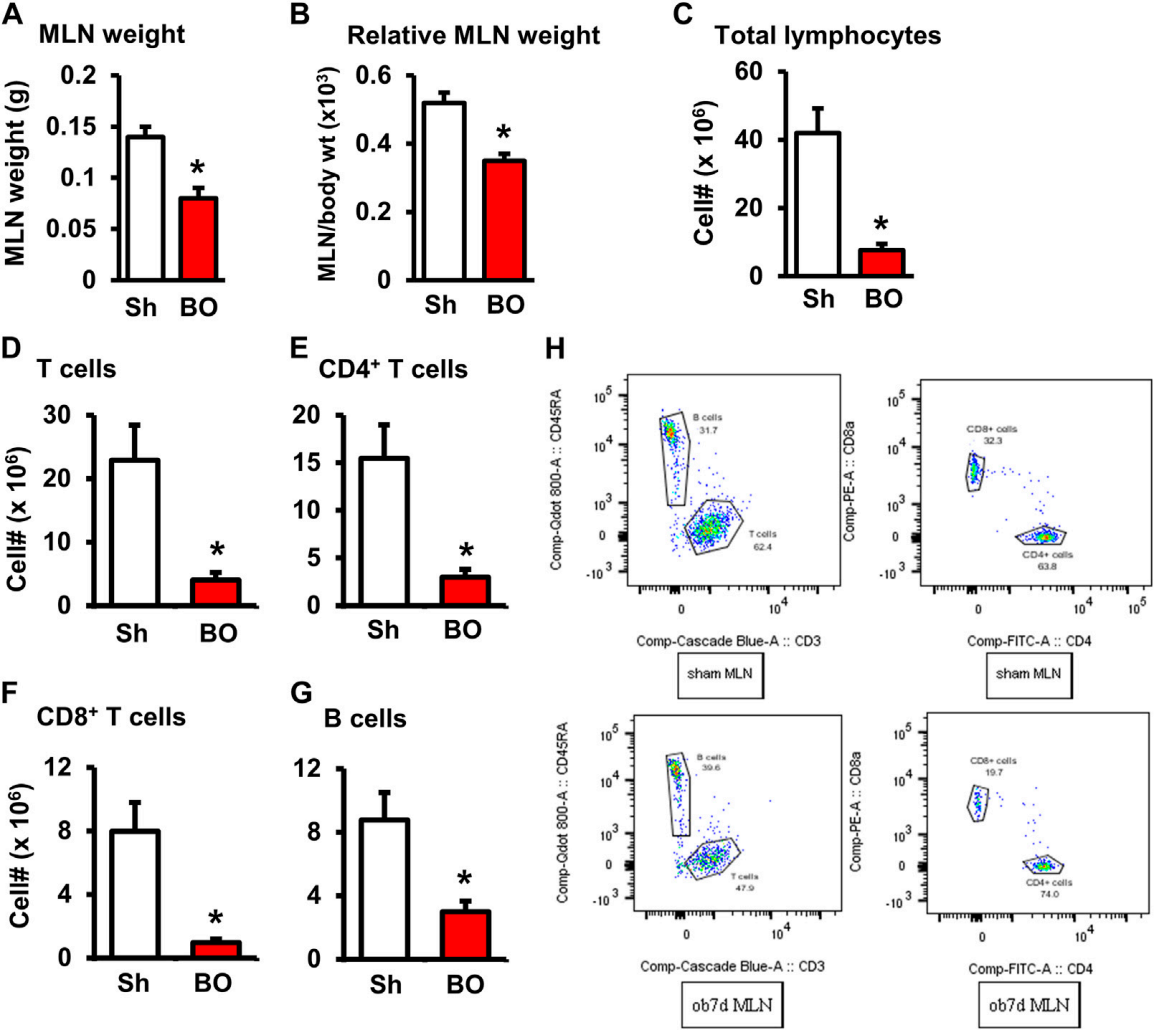
Effect of bowel obstruction on the mesenteric lymph node. **(A)** Weight of isolated mesenteric lymph node (MLN) in sham rats (Sh) and rats with bowel obstruction (BO) for 7 days. **(B)** Relative weight of the MLN [Ratio of MLN weight/body weight (x 10^3^)]. **(C)** Total live cells in the MLN. **(D)** Total T cells (CD3^+^); **(E)** CD4^+^ cells (CD3^+^/CD4^+)^; **(F)**. CD8^+^ cells (CD3^+^/CD4^+^); **(G)** B lymphocytes (CD45RA^+^). **(H)** Representative flow cytometry profiles for B and T lymphocytes in sham (upper left) and BO rats (down left), and CD4^+^and CD8^+^ cells in sham (upper right) and BO rats (down right). N = 6 per group. *p < 0.05 vs. sham. Sh, sham. BO, bowel obstruction.

**FIGURE 3:**
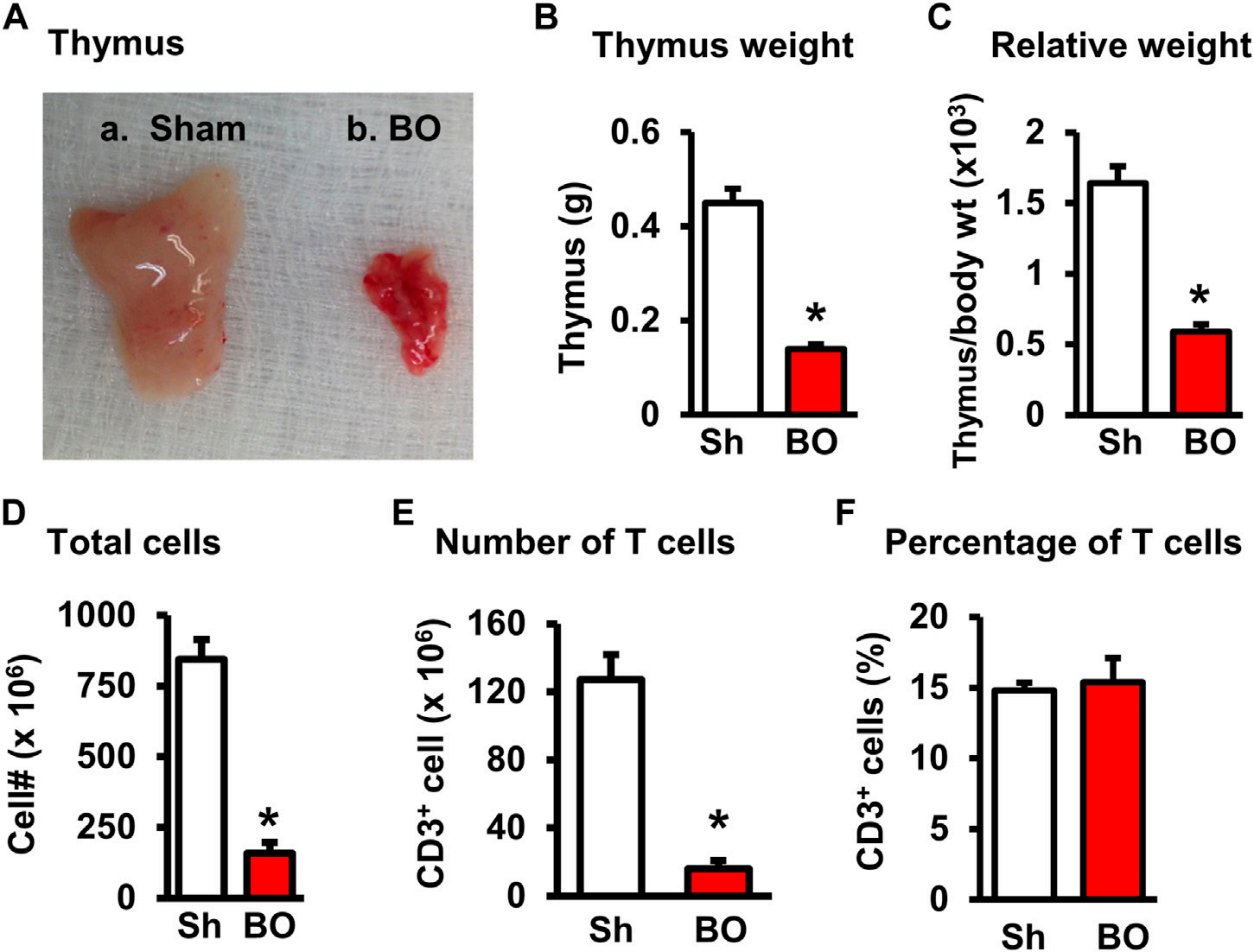
Effect of bowel obstruction on the thymus. **(A)** Isolated thymuses from a sham rat **(A)** and a rat with bowel obstruction for 7 days **(B)**. **(B)** Weight of thymus in Sh and BO rats. **(C)** Ratio of thymus weight/body weight (x 10^3^). **(D)** Total live lymphocytes. **(E)** Number of T cells (CD3^+^). **(F)** Percentage of T cells (CD3^+^) among all lymphocytes. N = 9 per group for B and **(C)** N = 5 or 6 per group in D, E, and **(F)** *p < 0.05 vs. sham. Sh, sham. BO, bowel obstruction.

The weight of peripheral lymphoid organs was significantly decreased in chronic bowel obstruction. Compared to sham, the weight of the spleen was decreased by 56.0% after 7 days of partial colon obstruction (0.70 ± 0.03 g in sham vs. 0.33 ± 0.04 g in BO, *p* < 0.01. N = 9) (Figures 1A,B). The body weight was also decreased in rats with BO. However, the body weight in rats with BO for 7 days was only 19.3% smaller than sham controls (284.27 ± 8.53 g in sham vs. 231.93 ± 8.10 g in BO, *p* < 0.01. N = 9). Overall, the relative weight of the spleen (spleen/body weight) was still significantly decreased in obstruction (Figure 1C). Compared to sham, the weight of MLN and the relative MLN weight (MLN/body weight) was also significantly reduced in BO rats (Figures 2A,B). Bowel obstruction also led to thymic atrophy. Compared to sham, the thymus weight decreased by 71.7% in rats with partial colon obstruction on day 7 (0.45 ± 0.03 g in sham vs. 0.14 ± 0.01 g in BO, *p* < 0.0001. N = 9) (Figures 3A–C). These data suggest that chronic BO leads to significant atrophy of primary and peripheral lymphoid organs.

**FIGURE 4:**
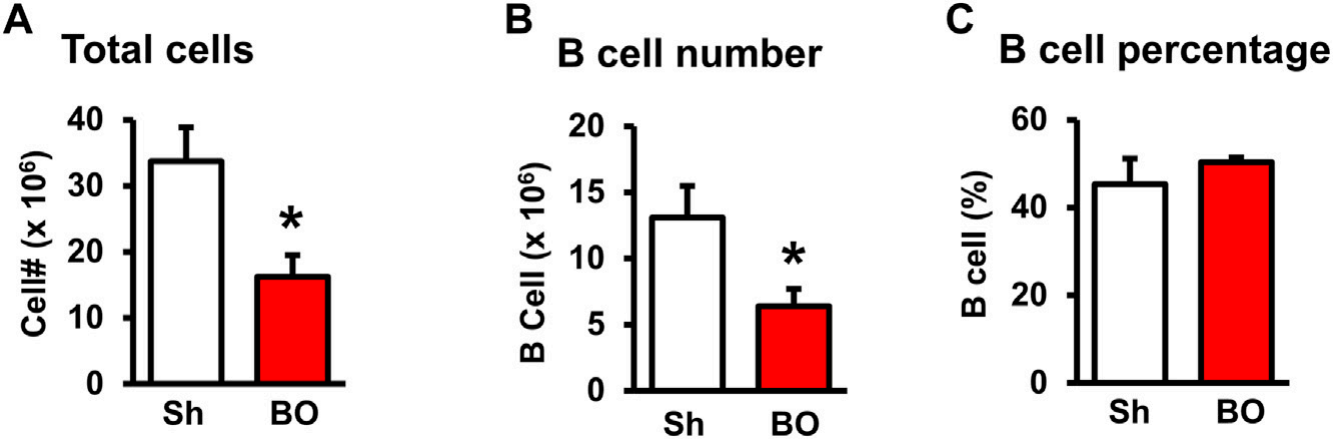
Effect of bowel obstruction on the bone marrow. **(A)** Total cells from sham rats (Sh) and rats with bowel obstruction for 7 days. **(B)** Number of B lymphocytes (CD45RA^+^). **(C)** Percentage of B lymphocytes (CD45RA^+^) among total lymphocytes. N = 5 or 6 per group. *p < 0.05 vs. sham. Sh, sham. BO, bowel obstruction.

We then counted the total lymphocytes in the lymphoid organs of sham and BO rats. Consistent with the weight changes, the total cell numbers of the spleen (Figure 1D), MLN (Figure 2C), thymus (Figure 3D), and bone marrow (Figure 4A) were all significantly reduced in bowel obstruction (*p* < 0.05 vs. sham).

### Effect of bowel obstruction on total T lymphocytes, CD4^+^ and CD8^+^ T cells in the lymphoid organs

To determine if obstruction affects lymphocyte subtypes in the lymphoid tissues, we performed flow cytometry to assess T cells in the thymus, and total, CD4^+^ and CD8^+^ T cells in the spleen, and MLN in sham control and rats with BO (7 days). Compared to sham, the number of total T cells was significantly decreased in obstruction in both the thymus (127.0 (±14.8) × 10^6^ vs. 22.2 (±5.0) × 10^6^, N = 7 or 8. *p* < 0.05) (Figure 3) and spleen (52.5 (±19.3) × 10^6^ vs. 10.8 (±1.3) × 10^6^, N = 7–9. *p* < 0.05) (Figure 1). Further subtype staining showed that the CD4^+^ T cells were significantly decreased in BO in both the spleen [39.9 (±9.1) × 10^6^ in sham vs. 17.2 (±4.5) × 10^6^ in BO, (N = 7–9. *p* < 0.05)] (Figure 1). The number of CD8^+^ T cells was also significantly decreased in the spleen (26.1 (±4.3) × 10^6^ vs. 8.5 (±1.5) × 10^6^) (All N = 7–9, *p* < 0.05) (Figure 1). The numbers of T lymphocytes, CD4^+^ and CD8^+^ T cells in the MLN were also all significantly decreased in BO, compared to sham control (Figures 2E–H). However, percentages of T cells among total lymphocytes, CD4^+^ and CD8^+^ cells among the total T cells were not significantly different between sham and BO groups in these lymphoid tissues (Figure 1F, Figure 2H, Figure 3F).

### Effect of bowel obstruction on B cells in the bone marrow and peripheral lymphoid organs

Staining for mature B cells (CD45RA^+^ cell) showed that the numbers of B cells in the spleen and MLN were significantly reduced in obstruction (Figures 1D,E and Figure 2G). The B cell number in the spleen was 43.2 (±5.0) × 10^6^ in sham, and 15.7 (±2.0) × 10^6^ in BO (*p* < 0.05). To determine if the reduction of B cells was due to loss of mature B cells in the peripheral lymphoid organs or suppression of B lymphopoiesis, we carried out a flow cytometry analysis of bone marrow cells from sham and BO rats. It was found that B cell numberer in the bone marrow was significantly reduced in obstruction [13.1 (±1.8) × 10^6^ in sham vs. 6.4 (±1.2) × 10^6^ in BO (N = 6 or 7. *p* < 0.05] (Figure 4B). Compared to sham, the percentage of B cells among total lymphocytes in the spleen, MLN, and bone marrow was not significantly changed in BO (Figure 1F, Figure 2H, Figure 4C).

### Role of corticosterone in BO-associated changes of lymphocytes in lymphoid organs

**FIGURE 5:**
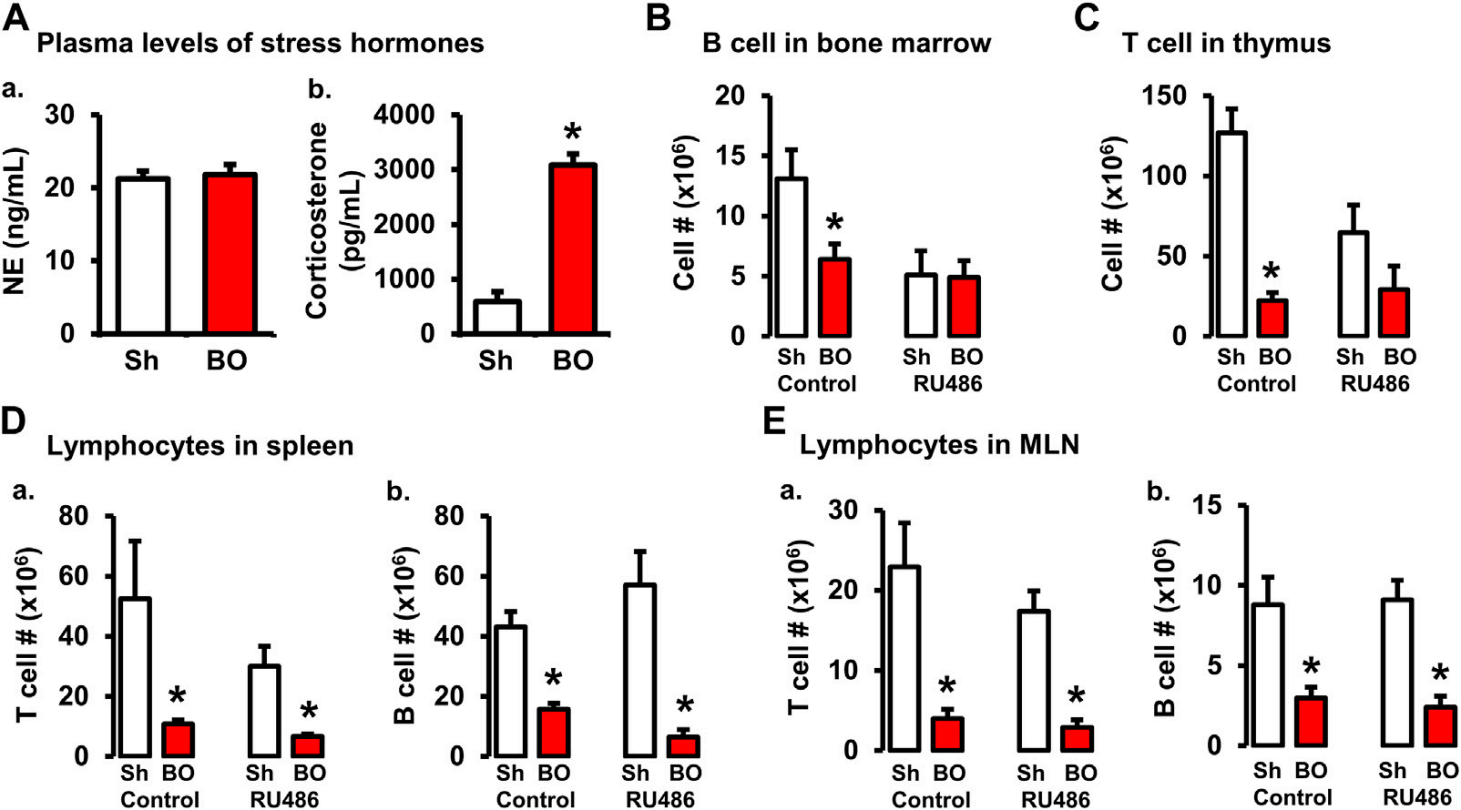
Role of corticosterone in BO-associated lymphopenia in lymphoid organs. **(A)** Plasma levels of norepinephrine **(A)** and corticosterone **(B)** in sham (Sh) and obstruction (BO) rats. N = 5 per group. *p ≤ 0.05 vs. sham. **(B)** Effect of corticosteroid receptor antagonist RU486 on B cell numbers in bone marrow in sham and BO rats. **(C)** Effect of RU486 on T cell numbers in thymus in sham and BO rats. **(D)** Effect of RU486 on T cells **(A)** and B cells **(B)** in the spleen in sham and BO rats. **(E)** Effect of RU486 on T cells **(A)** and B cells **(B)** in the MLN in sham and BO rats. N = 5 or 6 for B-E. *p ≤ 0.05 vs. sham in the treatment group.

BO, as a painful disorder, may increase stress hormone production (Choudhury et al., 2009). To determine if stress hormones are involved in BO-associated immune impairments, we first measured plasma norepinephrine (NE) and corticosterone, two major stress hormones, in sham and BO rats. The plasma NE levels were not significantly different between sham and BO rats (Figure 5A). However, plasma corticosterone level was significantly increased in BO rats (7 days) compared to sham controls (3,085.8 ± 179.0 ng/ml vs. 588.6 ± 179 ng/ml, *n* = 4, *p* < 0.05) (Figures 5A,B).

We then investigated if increased corticosterone contributed to immune impairments in BO by treating rats with corticosteroid receptor antagonist RU486. As shown in Figures 5B–E, RU486 treatment did not significantly attenuate BO-associated reduction of T cells and B cells in the peripheral lymphoid organs (i.e., spleen and MLN). However, the RU486 treatment significantly attenuated BO-induced reduction of T cells in the thymus (Figure 5C) and B cells in the bone marrow (Figure 5B), indicating that increased corticosterone in BO contributes to immune impairments.

### Role of gut microbiota in BO-associated changes of lymphocytes in lymphoid organs

**FIGURE 6:**
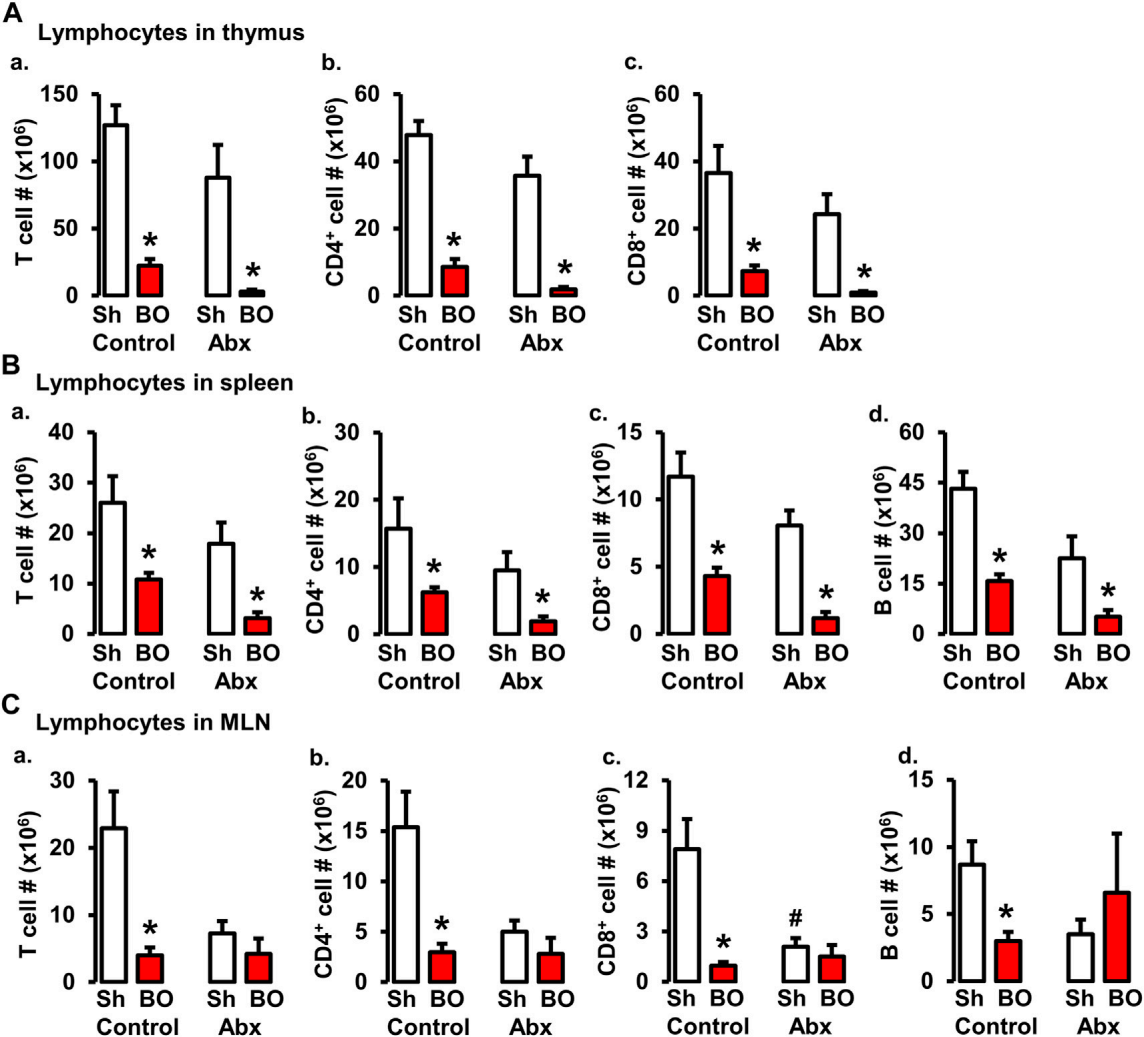
Role of gut microbiota in BO-associated lymphopenia in lymphoid organs. **(A)** Numbers of total T cells **(A)**, CD4^+^ cells **(B)**, and CD8^+^ cells **(C)** in the thymus in sham (Sh) and obstruction (BO) rats in control (no antibiotics treatment) and with antibiotics treatment (Abx). **(B)** Numbers of total T cells **(A)**, CD4^+^ cells **(B)**, CD8^+^ cells **(C)**, and B cells **(D)** in the spleen in Sh and BO rats in control and with Abx treatment. **(C)** Numbers of total T cells **(A)**, CD4^+^ cells **(B)**, CD8^+^ cells **(C)**, and B cells **(D)** in the MLN in Sh and BO rats in control and with Abx treatment. N = 5 or 6. *p ≤ 0.05 vs. sham in the treatment group. ^#^ *p* ≤ 0.05 vs. sham in the control group.

Previous studies suggest that gut microbiota plays a crucial role in lymphoid organ development and maturation (Kamada and Núñez, 2013; Cosola et al., 2021). Our own work has shown that bowel obstruction significantly alters gut microbiota compositions and diversity (Hegde et al., 2018). To determine if altered gut microbiota is responsible for BO-associated immune impairments, sham and BO rats were gavage treated daily with a cocktail of antibiotics (Hegde et al., 2018) to deplete gut microbiota. The cellularity in the lymphoid organs was determined. Antibiotic treatment drastically decreased total gut microbiota by more than 1,500-fold in sham and BO rats (Hegde et al., 2018). Depletion of gut microbiota had a trend to decrease lymphocyte numbers in the spleen and MLN, and significantly decreased CD8^+^ cells in the MLN of sham rats (Figure 6). More importantly, depletion of gut microbiota prevented BO-associated reduction of T and B lymphocytes in the MLN (Figure 6C), though it had little effect on BO-induced immune impairments in the spleen and thymus (Figures 6A,B).

### Lumen distention induced mechano-sensitive expression of osteopontin in the colon and increased plasma osteopontin levels in bowel obstruction

**FIGURE 7:**
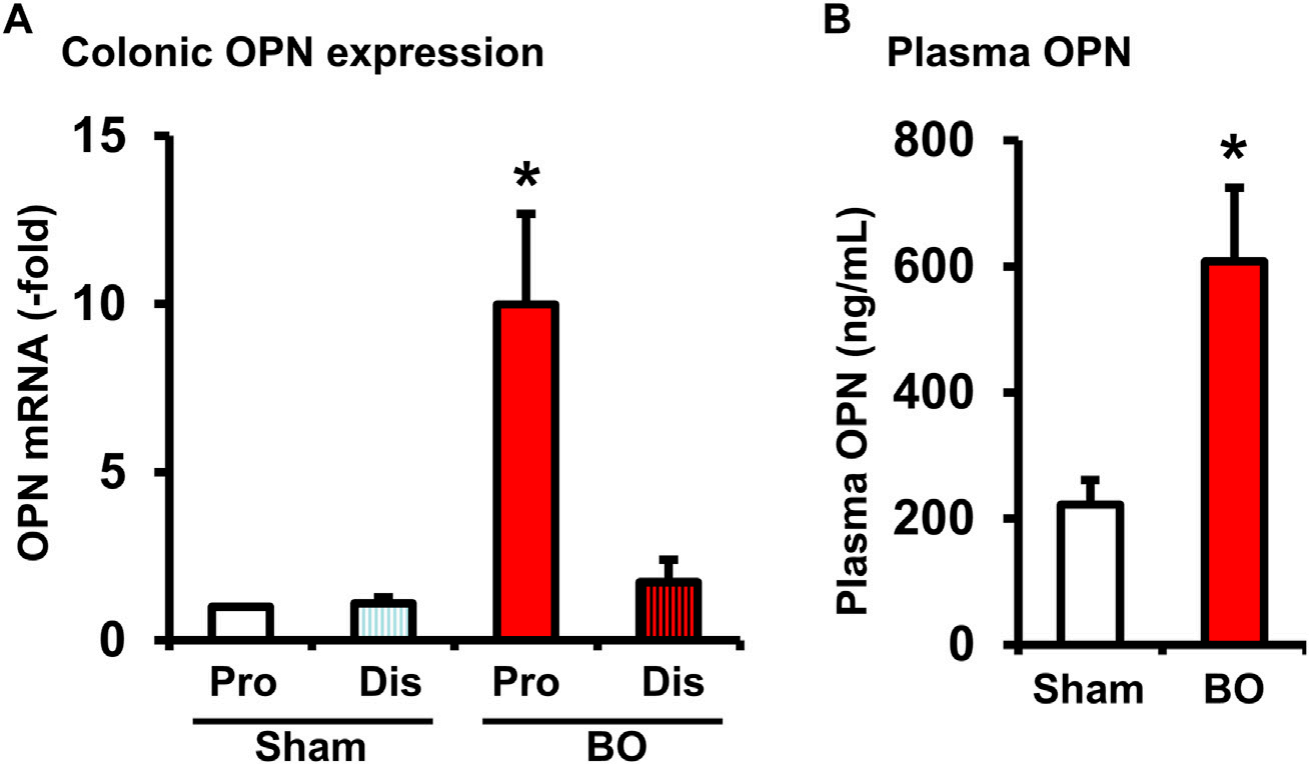
Osteopontin expression in the colon **(A)** and plasma OPN levels **(B)** during bowel obstruction. Rats with sham operation and bowel obstruction (BO) for 7 days were euthanized for collections of colon tissues and blood. The muscularis externa tissues of the colonic segments 2 cm proximal (Pro) and 1 cm distal (Dis) to the site of obstruction band were taken for real-time qPCR study. Left ventricle blood was taken for preparation of plasma samples and EIA measurements of OPN. N = 5 or 6. *p ≤ 0.05 vs. Sham.

OPN is a multifunctional protein (Icer and Gezmen-Karadag, 2018) and was found to play a role in acute thymus atrophy (Wang et al., 2007; Wang et al., 2009). OPN gene expression is also highly sensitive to mechanical stretch (Denhardt et al., 2001; Wongkhantee et al., 2007). We found that OPN mRNA expression was increased significantly by nearly 10-fold in the muscularis externa of the distended colon segment proximal to obstruction but not in the non-distended segment distal to obstruction (Figure 7A). We further determined the OPN levels in the plasma of sham and BO rats (day 7). Compared to sham control (222.62 ± 39.01 ng/ml), plasma OPN level in BO rats (608.80 ± 116.58 ng/ml) was significantly increased (*p* < 0.05. N = 4 rats in sham and 6 in BO) (Figure 7B). These data suggest that mechanical distention dramatically induced OPN expression in the colon, which in turn led to a significant increase of OPN levels in the blood in BO.

### Obstruction-initiated changes of T and B lymphocytes in wild-type and OPN^−/−^ animals.

**FIGURE 8:**
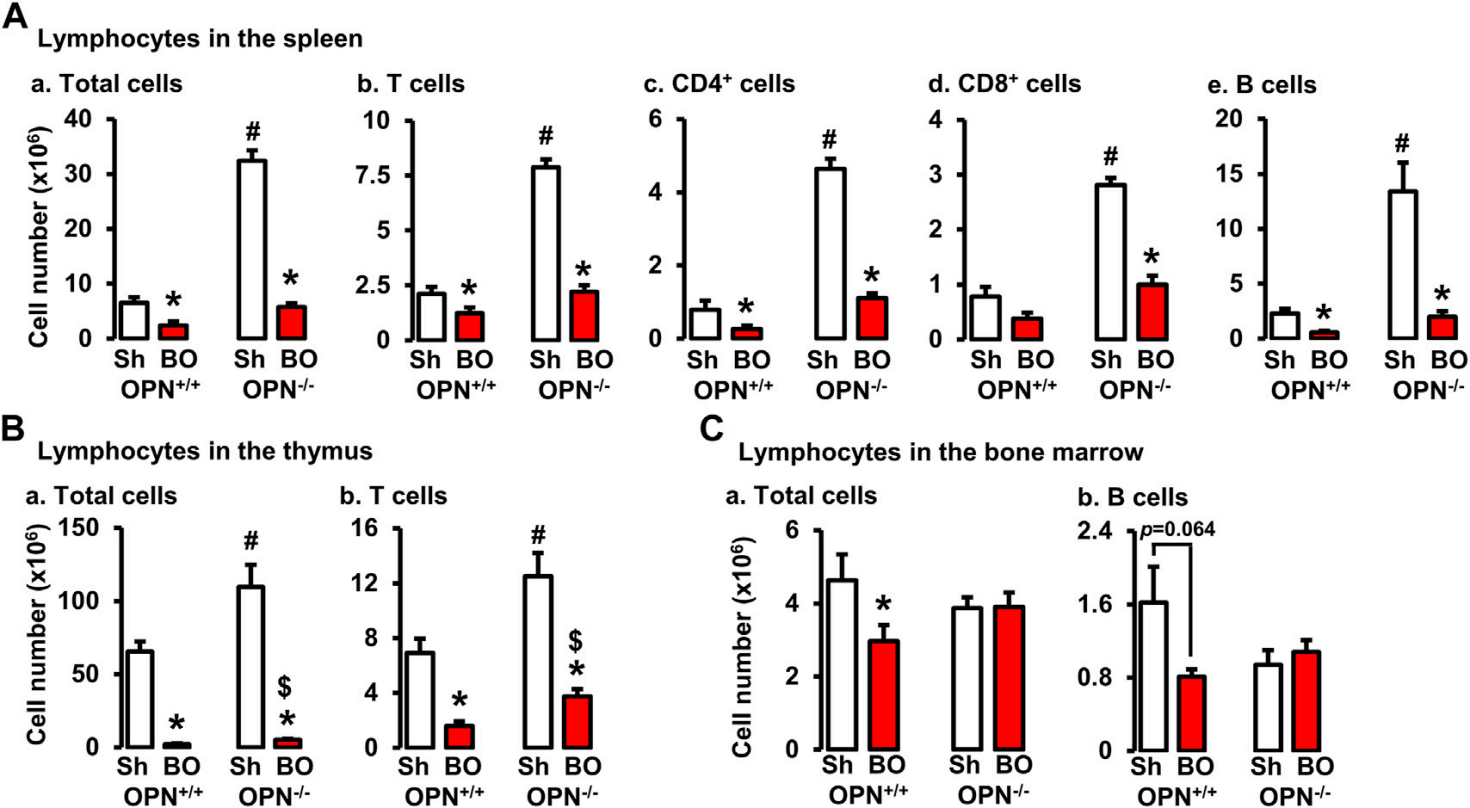
Effects of bowel obstruction on specific lymphocytes in lymphoid organs in wild-type (OPN+/+) and osteopontin knock out (OPN−/−) mice. **(A)** Numbers of total live lymphocytes **(A)**, T cells **(B)**, CD4^+^ cells **(C)**, CD8^+^ cells **(D)** and B cells **(E)** in the spleen of OPN^+/+^ and OPN^−/−^ mice with sham operation (Sh) and obstruction (BO, 4 days). **(B)** Numbers of total live lymphocytes **(A)** and T cells **(B)** in the thymus of OPN^+/+^ and OPN^−/−^ mice with sham operation (Sh) and obstruction (BO, 4 days). **(C)** Numbers of total live lymphocytes **(A)** and B cells **(B)** in the bone marrow of OPN^+/+^ and OPN^−/−^ mice with sham operation (Sh) and obstruction (BO, 4 days). N = 6 or 7. *p ≤ 0.05 vs. sham of the group. ^#^ *p* ≤ 0.05 vs. sham of OPN^+/+^. ^$^ *p* ≤ 0.05 vs. BO of OPN^+/+^.

To determine if OPN plays a role in the impairment of T and B lymphopoiesis and maintenance in bowel obstruction, we quantitated total lymphocyte numbers and analyzed T and B cells by flow cytometry in the spleen, thymus, and bone marrow (Figures 8A–C) in wild-type (OPN^+/+^) and OPN gene knockout (OPN^−/−^) mice. BO led to a significant reduction of total cells and T lymphocytes in the thymus, and total, CD4^+^ and CD8^+^ T cells in the spleen not only in wild-type mice, but also in OPN^−/−^ mice (Figures 8A,B). However, BO-associated reduction of total cells and T lymphocytes in the thymus was partially recovered in OPN^−/−^ mice, compared to wild-type BO mice (N = 6 or 7. *p* < 0.05) (Figure 8B). In the bone marrow, BO led to reduction of total lymphocytes and B cells only in the wild-type mice, but not in the OPN^−/−^ mice (Figure 8C). Finally, the cell numbers of total lymphocytes and T cells in the thymus, and total, CD4^+^ and CD8^+^ T cells in the spleen of sham OPN^−/−^ mice were all significantly greater than that of sham wild-type mice (Figures 8A,B). This data suggests that OPN may play an important role in normal T lineage lymphopoiesis and is involved in the suppression of T and B lymphopoiesis in bowel obstruction (Figure 8).

## Discussion

Our study in the rodent model of partial colon obstruction demonstrates that persistent bowel distention leads to atrophy with decreased cellularity in the primary and secondary lymphoid organs. The atrophy in the lymphoid organs was a result of significantly decreased numbers of lymphocytes in these organs. We found that the thymus and spleen weights are decreased by 50%–70% in rats with partial colon obstruction for 7 days. The numbers of total T lymphocytes (CD3^+^) in the thymus and total, CD4^+^, and CD8^+^ T cells in secondary lymphoid organs including spleen and MLN are all dramatically decreased in obstruction. The B lymphocytes in the bone marrow and secondary lymphoid organs are also significantly reduced in BO. These results suggest that bowel obstruction may be considered a novel model of lymphoid organ atrophy and lymphoid depletion. Suppression of lymphocyte generation and maintenance in the lymphoid organs was found to be associated with septic shock (Felmet et al., 2005; Girardot et al., 2017), and may contribute to the development of infections, enterocolitis, sepsis, and immune impairments encountered in chronic bowel obstruction and Hirschsprung’s disease (Levine and Price, 1994; Gosain et al., 2017).

Immune impairments have been reported in chronic obstructive bowel disorders such as intestinal pseudo-obstruction (Forchielli et al., 1997) and intestinal atresia (Notarangelo, 2014). The serum immunoglobulin gamma was found decreased in patients with chronic intestinal pseudo-obstruction (Forchielli et al., 1997). In fact, nearly 78% of patients with the chronic functional obstruction had various immunoglobulin deficiencies, indicating an impaired development or maintenance of B lymphocytes in chronic OBD. Intestinal atresia is a relatively rare condition in newborns. The etiology of the condition is not clear. However, atresia always leads to significant distention of the bowel segments proximal to the occlusion. Clinical studies have shown that intestinal atresia patients are associated with significant immune dysfunction and immune organ atrophy (Notarangelo, 2014). As mechanical bowel obstruction is an acute abdomen and is usually managed in an urgent manner, there has been no report on the status of immune organs and immune function in the patients with acute bowel obstruction. However, our preliminary clinical laboratory data analysis found that the peripheral lymphocyte count is lower in patients with bowel obstruction compared to normal control subjects (Shi XZ, unpublished observation). Interestingly, these patients had normal or slightly higher neutrophil counts. The neutrophil-lymphocyte ratio is higher than normal. This observation is consistent with an earlier report in mouse megacolon model of Hirschsprung’s disease, which showed that common lymphocyte progenitor populations, but not hematopoietic stem cells, were reduced in the bone marrow in animals with megacolon (Frykman et al., 2015). The megacolon mice also demonstrated thymic atrophy and severe lymphoid depletion. Thus, it appears that chronic bowel obstruction may only affect lymphopoiesis, but not other hematopoietic pathways. However, it will be interesting to further determine if bowel obstruction impacts innate immunity, and if so, whether innate immunity changes contribute to enterocolitis, infection, and sepsis in OBD.

Hirschsprung’s disease is a chronic OBD, as a partial obstruction in the aganglionic distal colon leads to bowel distention in the proximal colon or even small intestine (Frykman et al., 2015; Gosain et al., 2015; Gosain et al., 2017). Hirschsprung’s associated enterocolitis is the most common and severe complication in children with Hirschsprung’s disease (Gosain et al., 2017). Recent studies detected significant thymic involution, splenic lymphopenia and suppression of T and B lymphopoiesis in murine models of Hirschsprung’s disease either with endothelin receptor B deletion (Gosain et al., 2015) or endothelin 3-null mice (Frykman et al., 2015). The Hirschsprung’s disease mouse models demonstrated a remarkable colon obstruction. Interestingly, the immune abnormalities occurred earlier than Hirschsprung’s associated enterocolitis in these mice. Several genes and gene loci including genes encoding endothelin 3 or endothelin receptor B are found to be responsible for aganglionosis, a developmental abnormality of the enteric nervous system, in Hirschsprung disease. Aganglionosis in the distal bowel causes occlusion of the distal bowel and consequently remarkable distention of the bowel prior to the occlusion. As the immune abnormalities were discovered in the local gut immune organs, spleen, and thymus in the murine model of Hirschsprung’s disease with either endothelin receptor B deletion or in endothelin 3-null mice, investigators suspected that endothelin 3, endothelin receptor B, or the enteric nervous system may be involved in immune regulation (Frykman et al., 2015; Gosain et al., 2015). However, our study found that the same immune phenotype (i.e. thymic involution, splenic lymphopenia and suppression of T and B lymphopoiesis) occurs in the model of mechanical bowel obstruction. This demonstrates that the root cause of immune abnormalities in OBD is bowel distention.

As BO-associated atrophy and lymphopenia in lymphoid organs represent a new immune phenotype, we sought to uncover its pathophysiological mechanisms. Osteopontin (OPN) was found to be crucial in mediating thymic or spleen atrophy and immune impairment (Wang et al., 2007; Wang et al., 2009). In a model of hindlimb unloading model, Wang et al. found that the thymus and spleen were significantly smaller in the wild-type mice with the treatment of hindlimb unloading, and serum OPN was increased in these mice (Wang et al., 2007). However, in OPN^−/−^ mice, the same treatment of hindlimb unloading did not lead to lymphoid organ atrophy. It was implied that OPN might mediate lymphoid organ atrophy. In our model, partial colon obstruction is associated with lymphoid organ atrophy and increased plasma OPN levels. Furthermore, we identified that the increased serum OPN in our model was due to mechano-sensitive expression of OPN in the distended colon proximal to obstruction, as the expression of OPN in the non-distended colon segment distal to obstruction was not increased. We found that BO still led to a dramatic decrease of total lymphocytes and T and B cells in the thymus and spleen not only in the wild-type mice but also in the OPN^−/−^ mice. However, the number of total T cells in the thymus in the OPN^−/−^ animals was significantly greater than in wild-type animals in obstruction. Moreover, BO-associated suppression of lymphocyte generation in the bone marrow only occurred in the wild-type mice, but not in the OPN^−/−^ mice. Interestingly, the cell numbers of total lymphocytes and T cells in the thymus and spleen of sham OPN^−/−^ mice were all significantly greater than that of sham wild-type mice. Together, these data suggest that OPN may not only be involved in BO-associated disruption of T and B lineages lymphopoiesis, but also play an important regulatory role in normal T lineage lymphopoiesis. The latter finding is consistent with previous reports that OPN may be a T-lymphocyte suppressor factor (Weber and Cantor, 1996).

We found that stress hormone corticosterone was increased in obstruction, indicating that BO rats might be distressed. As corticosterone is known to affect immune functions (Wang et al., 2007; Costa-Pinto and Palermo-Neto, 2010), we then determined if corticosterone is involved in the BO-associated reduction of lymphocytes in the primary and secondary lymphoid organs. Treatment with corticosteroid receptor antagonist RU486 indeed significantly attenuated BO-associated reduction of lymphoid development in the primary lymphoid organs bone marrow and thymus. However, corticosteroid receptor inhibition does not affect the cellularity changes by BO in the peripheral lymphoid organs, such as the spleen and MLN.

Our study shows that the gut microbiota may contribute to the maturation of lymphocytes in the gut-associated lymphoid tissues (GALT), as depletion of the gut microbiota had the trend to reduce the total, T and B cells in the MLN. More importantly, BO-associated reduction of lymphocytes in the MLN was blocked when gut microbiota was depleted. Interestingly, the effect of gut microbiota on immune development appears to be a local effect, as depletion of gut microbiota did not affect BO-associated immune impairments in the spleen or primary lymphoid organs i.e., thymus and bone marrow. Our results suggest that microbiota dysbiosis in chronic obstruction contributes to BO-associated lymphopenia in MLN.

Our mechanistic studies suggest that multiple factors are involved in BO-induced lymphoid depletion in the lymphoid organs. Stress hormone corticosterone and mechano-sensitive production of OPN in the bowel may contribute to the suppression of lymphopoiesis in the primary organs, whereas BO-associated microbiota changes are responsible for local lymphopenia in the GALT. It is noticeable that none of the interventions affects BO-associated lymphopenia in the spleen. This may be due to the fact that we only studied one time point, i.e., 7 days in rats or 4 days in mice after induction of obstruction. Although the effect of RU486 and OPN deficiency on primary lymphoid organs may be detected on day 7 or day 4 of obstruction, it may take longer to detect any changes in the spleen and other peripheral lymphoid organs. In addition, mechanism(s) other than what we have explored may also be involved in the development of immune abnormality by bowel obstruction. For instance, IL-6 and leukemia inhibitory factor are among the cytokines and pro-inflammatory mediators involved in thymic atrophy and lymphoid depletion (Sempowski et al., 2000; Gruver and Sempowski, 2008). Our previous studies showed that gene expression of IL-6 and leukemia inhibitory factor in addition to OPN are highly sensitive to mechanical stress in the colon (Lin et al., 2014; Hegde et al., 2018; Lin et al., 2020; Geesala et al., 2021). Further study is warranted to better understand the pathophysiological role of mechanical stress-altered gene expression in the gut in lymphoid depletion and immune impairments in OBD.

It is noteworthy that although the numbers of total lymphocytes, and B and T cells in the primary and peripheral lymphoid tissues are markedly reduced by BO, the percentages of B and T cells among total lymphocytes are not changed. This suggests that BO may not affect differentiation of individual B cells or T cells, or subtypes of T lymphocytes, rather mainly suppressing lymphopoiesis in the thymus and bone marrow. Reduction of lymphocyte generation in the primary lymphoid organs will lead to decreased cell numbers of total and T and B lymphocytes in the peripheral lymphoid tissues. Further study is needed to better understand the mechanisms of BO-associated suppression of lymphopoiesis. However, our exploratory studies suggest that corticosterone and OPN are involved in the mechanisms as corticosteroid antagonist or OPN deletion attenuated BO effect on lymphopoiesis. Previous studies found that corticosteroid causes apoptosis of pre-B and mature B cells (Alnemri et al., 1992; Frykman et al., 2015), and OPN suppresses T cell development (Weber and Cantor, 1996).

Taken together, our study shows that bowel obstruction leads to profound suppression of lymphopoiesis and maintenance of lymphocyte repertoires in the lymphoid organs. Stress hormone corticosterone and mechano-sensitive production of OPN in the colon contribute to the suppression of lymphopoiesis in the primary organs, whereas BO-associated microbiota changes may be responsible for lymphopenia in the lymph nodes in the gut.

## Data Availability

The raw data supporting the conclusion of this article will be made available by the authors, without undue reservation.

## Abbreviations

BO, bowel obstruction; COX, cyclooxygenase; ME, muscularis externa; M/S, mucosa/submucosa; GALT, gut-associated lymphoid tissues; MLN, mesenteric lymph node; OPN, osteopontin; OBD, obstructive bowel disorders; PG, prostaglandin; PP, Peyer’s patch; SMC, smooth muscle cells.
